# Determining the scope of attacks on health in four governorates of Syria in 2016: Results of a field surveillance program

**DOI:** 10.1371/journal.pmed.1002559

**Published:** 2018-04-24

**Authors:** Rohini J. Haar, Casey B. Risko, Sonal Singh, Diana Rayes, Ahmad Albaik, Mohammed Alnajar, Mazen Kewara, Emily Clouse, Elise Baker, Leonard S. Rubenstein

**Affiliations:** 1 School of Public Health, University of California, Berkeley, California, United States of America; 2 Department of International Health, Johns Hopkins Bloomberg School of Public Health, Baltimore, Maryland, United States of America; 3 Department of Medicine, University of Massachusetts Medical School, Worcester, Massachusetts, United States of America; 4 Center for Public Health and Human Rights, Johns Hopkins Bloomberg School of Public Health, Baltimore, Maryland, United States of America; 5 Syrian American Medical Society, Gaziantep, Turkey; 6 Physicians for Human Rights, New York, New York, United States of America; Massachusetts General Hospital, UNITED STATES

## Abstract

**Background:**

Violent attacks on and interferences with hospitals, ambulances, health workers, and patients during conflict destroy vital health services during a time when they are most needed and undermine the long-term capacity of the health system. In Syria, such attacks have been frequent and intense and represent grave violations of the Geneva Conventions, but the number reported has varied considerably. A systematic mechanism to document these attacks could assist in designing more protection strategies and play a critical role in influencing policy, promoting justice, and addressing the health needs of the population.

**Methods and findings:**

We developed a mobile data collection questionnaire to collect data on incidents of attacks on healthcare directly from the field. Data collectors from the Syrian American Medical Society (SAMS), using the tool or a text messaging system, recorded information on incidents across four of Syria’s northern governorates (Aleppo, Idleb, Hama, and Homs) from January 1, 2016, to December 31, 2016. SAMS recorded a total of 200 attacks on healthcare in 2016, 102 of them using the mobile data collection tool. Direct attacks on health facilities comprised the majority of attacks recorded (88.0%; *n* = 176). One hundred and twelve healthcare staff and 185 patients were killed in these incidents. Thirty-five percent of the facilities were attacked more than once over the data collection period; hospitals were significantly more likely to be attacked more than once compared to clinics and other types of healthcare facilities. Aerial bombs were used in the overwhelming majority of cases (91.5%). We also compared the SAMS data to a separate database developed by Physicians for Human Rights (PHR) based on media reports and matched the incidents to compare the results from the two methods (this analysis was limited to incidents at health facilities). Among 90 relevant incidents verified by PHR and 177 by SAMS, there were 60 that could be matched to each other, highlighting the differences in results from the two methods. This study is limited by the complexities of data collection in a conflict setting, only partial use of the standardized reporting tool, and the fact that limited accessibility of some health facilities and workers and may be biased towards the reporting of attacks on larger or more visible health facilities.

**Conclusions:**

The use of field data collectors and use of consistent definitions can play an important role in the tracking incidents of attacks on health services. A mobile systematic data collection tool can complement other methods for tracking incidents of attacks on healthcare and ensure the collection of detailed information about each attack that may assist in better advocacy, programs, and accountability but can be practically challenging. Comparing attacks between SAMS and PHR suggests that there may have been significantly more attacks than previously captured by any one methodology. This scale of attacks suggests that targeting of healthcare in Syria is systematic and highlights the failure of condemnation by the international community and medical groups working in Syria of such attacks to stop them.

## Abstract 2

Rohini Haar and colleagues use a standardized reporting tool to verify field reports and count attacks on medical providers and facilities in a region of northern Syria

## Author summary

**Why was this study done?:**

- There has been no systematic and consistent means to document attacks on health facilities and health workers under international humanitarian law.
- This method differs from other methods by relying on definitions grounded in international humanitarian law and field reporting by dedicated data collectors.
- This study was done to quantify how many attacks on healthcare there have been in northern Syria, details of the attacks, risk factors for attack, and consequences.

**What did the researchers do and find?:**

- Data collectors in the field reported information on attacks on hospitals, transports, healthcare workers, and patients.
- A subset of these incidents (51%) were recorded on a standardized reporting tool using a mobile phone application.
- We found that there were more than 200 attacks on healthcare in 2016 in four northern governorates.
- In total, 112 health workers and 185 patients were killed in these incidents, and 176 of the attacks were on hospitals and other healthcare facilities.
- We compared data to another database maintained by PHR to show that no one methodology is capturing all of the incidents that have occurred.

**What do these findings this mean?:**

- These numbers shed light on the extent of attacks on civilian health infrastructure and personnel in Syria.
- The study suggests that the utilization of a standard reporting tool can contribute to the depth and quality of the reported data, especially in capturing incidents that may not be found using media data.
- It also suggests that this method requires intensive training and support resources for successful implementation.
- The comparison between SAMS and PHR databases suggests that multiple methods of tracking attacks may be needed to identify all incidents.
- The study also highlights the need for more research and advocacy towards accountability and stopping the attacks.

## Introduction

Acts of violence against healthcare facilities, transports, medical personnel, and patients are frequent but underreported in armed conflict [1–4]. Clinics, hospitals, private medical offices, and transports such as ambulances and supply trucks have been bombed, looted, blocked, or occupied worldwide [5–7]. Healthcare personnel and patients have been arrested, killed, maimed, tortured, interrogated, and blocked from receiving or providing care. Even bombings and shelling of hospitals sometimes go unreported, and other attacks, such as looting, obstructing passage at checkpoints, and threats to healthcare workers are usually not systematically tracked [8,9]. Attacks on health workers, facilities, and transports, and on the wounded and sick, violate international human rights law and international humanitarian law [10–12]. Recent resolutions from the United Nations (UN) Security Council and the UN General Assembly have reiterated the vital importance of protecting health during conflict and the need for data to track attacks [13,14]. It is well recognized, however, that no systematic data collection on attacks on healthcare services in conflicts has existed, though the World Health Organization (WHO) is beginning such an effort in 11 countries in 2017 based on a mandate from the World Health Assembly in 2012 [15,16]. Where data is collected on such attacks, definitions of what constitutes an attack vary considerably, and in many cases, only secondary data is collected [4].

Since the Syrian conflict began in 2011, an unprecedented number of attacks on hospitals and health workers has been reported [17–22] using various methods and approaches[21,23,24] and with different results. Physicians for Human Rights (PHR), a nonprofit human rights organization based in New York, has developed the most comprehensive publicly available registry and mapping of attacks on health facilities in Syria and has documented and verified more than 465 instances of attacks on healthcare facilities since March 2011 [25,26]. PHR collects data only on health facilities, excluding attacks on patients, medical personnel other than those killed or injured in facility attacks, and obstructions of access. It uses a methodology that examines press reports, social media posts, and other open-source information to corroborate reported attacks on healthcare facilities but does not collect data directly on the ground [24]. WHO and the UN Health Cluster for northern Syria, located in Turkey, have reported attacks on health services in Syria and elsewhere using reports submitted by nongovernmental organizations (NGOs) and other partners and verifying them (the Syrian American Medical Society [SAMS] is part of the UN Health Cluster) [23,27]. Medecins Sans Frontiers [28], the Syrian Network for Human Rights [29], and the Violations Documentation Center [30] also make efforts to report on attacks using different methods but without common definitions [31–33].

There is widespread recognition that many attacks are not reported due to ongoing conflict, difficulty in accessing the site to gain quality information, or lack of clarity on what constitutes an attack [34]. Furthermore, when attacks are documented, reports often lack information about the extent of damage, the number of healthcare personnel and patients killed and/or injured, and operational status. Because of the use of varying definitions, some methods do not capture threats against and arrests of health workers, restrictions on patients’ access to care, and other incidents that violate the provisions of international law demanding respect for and protection of health services. SAMS, with assistance from our research team, runs a unique program enlisting local data collectors in regions of Syria where it supports healthcare services to identify and characterize incidents in detail. Data from this program have been used to inform local and international advocacy efforts[35] and are shared as well with WHO’s health cluster for northern Syria.

This study sought to implement and test a method of collecting field data on the scope of and details about the attacks based on direct field reporting using questions centered on international human rights and humanitarian law. We also aimed to identify any characteristics that increase or mitigate vulnerability of healthcare facilities. We hoped to use this opportunity to evaluate the utility of a standardized field survey tool for documenting these attacks. To contextualize this documentation system, we aimed to compare the data to PHR’s database that uses media reports to track attacks on hospitals.

## Methods

We developed a standardized reporting questionnaire to document attacks on health in Syria based on a previously validated survey for tracking attacks on healthcare in Myanmar and grounded in the requirements of and definitions from international human rights and humanitarian law [36]. These laws demand that in times of armed conflict, combatants respect and protect the wounded and sick as well as health facilities, health workers, and transports; allow the flow of humanitarian aid; and refrain from punishing persons engaged in medical work in accordance with their ethical duties [11]. The questionnaire was adapted for use in Syria, using domains for attacks on facilities, health workers, transports, and patients, and underwent numerous iterations based on pilot data and feedback on accuracy, clarity, and conciseness from within the research team, as well as from data collectors and SAMS data managers based in Gaziantep, Turkey.

The questions covered attacks on four domains based on the requirements of law: (1) healthcare facilities and other settings where medical services could be provided (medical offices, laboratories, clinics, hospitals, forensic centers, and blood banks); (2) healthcare transports, including ambulances and vehicles carrying supplies and equipment; (3) healthcare personnel, including physicians, nurses, allied health professionals, and others providing clinical services; and (4) patients, both those that are already under care and those that seek care. The questionnaire also requested detailed information about an incident, including information about the victims; the perpetrator(s); date, time, and location; intentionality; type(s) of facilities; descriptions of the attack and type(s) of violence and weapon(s); damage inflicted; impact on functionality and service capacity; injuries; and deaths among patients and healthcare personnel, as well as narrative and image fields (the data collection tool is available from authors upon request). The questionnaire was translated into Arabic by the research team and verified by at least two other native Arabic speakers to ensure accuracy and clarity but, given resource limitations, was not formally back-translated. Revisions were discussed and made by consensus.

The questionnaire was uploaded to a computer-based data collection application developed by the Magpi Mobile Data Service (Magpi) [37] and further edited for use on the mobile platform. The application allowed offline entry and GPS stamping of the response location. It also permits customizable questionnaires that use skip logic and dropdown lists for ease of use and to help ensure reliability and standardization (S1 Fig). Data collectors in the field in northern Syria were able to use the Magpi application to answer the questions via mobile phone, and data managers in Turkey and the United States could view real-time reports based on the responses on computers as well as mobile devices. The Magpi application-based questionnaire (referred to as the “tool”) was chosen for its data security features, ability for offline entry of responses, the ease of mobile incident documentation, and real-time uploading and analysis of the data.

### Data collection

SAMS has documented attacks on health since 2014 in four northern governorates of Syria where it supports health services: Aleppo, Idleb, Homs, and Hama. These governorates have been the site of intense violence since the beginning of the war, with frequent attacks on civilian infrastructure, including health services [38]. The reporting tool was made available to data managers working for SAMS in Gaziantep, which hired data collectors and oversaw data collection since mid-2015. SAMS hired eight individuals who live in both rural and urban regions of Syria and represented all areas of these four governorates. Data collectors were recruited by SAMS field offices in each governorate based on their geographical knowledge, communication skills, and technical aptitude related to data collection. The data collectors were authorized to work by local health directorates. Data collectors were trained by the research team through its office in Gaziantep, Turkey. Training topics included mobile reporting (using the tool on smart phones); basic concepts of the law of protection and respect for healthcare; ethical issues in data collection; security issues; and self-care. Because of the security situation inside Syria and the difficulty of traveling across the Syria–Turkey border, data collectors engaged in online refresher trainings with the research team with ongoing communication and support with SAMS data managers in Gaziantep as well as field officers in their respective governorates in Syria. Periodic review of the database by the research team informed discussions with the SAMS officers on addressing any problems with the data collection mechanism.

Each data collector was responsible for reporting on attacks in a specified geographical range where they lived. Data collectors identified incidents through a variety of methods, including mobile communication with fellow staff in SAMS-supported hospitals and the large network of health service providers in Syria, as well as the SAMS data managers who were notified of incidents directly by health workers, hospital administrators, witnesses, or through the health cluster.

Once an incident was identified, the regional data collector visited the site of attack and sought out hospital staff, administrators, and witnesses to corroborate the incident and complete the survey. Data collectors were trained to only report on incidents after the attack had completely ceased and the area was secured. The data collectors interviewed informants using the reporting tool question fields and captured photographs and video of the incident, if relevant. They communicated basic information about the incident with SAMS data managers through a secure messaging system and were encouraged to characterize the full details of the incident using the Magpi program. SAMS data managers in Gaziantep oversaw the documentation process on each report and clarified any incomplete information with the data collectors. Data managers were responsible for verifying incidents with the health cluster network and with their personnel inside Syria. SAMS corroborated all incidents and details with multiple sources, including reports from other health staff, witnesses, and the health cluster, as well as video and photographs. Discrepancies in the data were reviewed by data collectors and data managers, and further investigation of those incidents was conducted using the resources described above.

### Data analysis

The research team conducted analysis on the data collected using Magpi (51% of the incidents), as well as incident reports received via other forms of communication, such as messaging applications, emails, and personal communication. We attempted to understand trends in the data and study any variables that may have posed increased vulnerability for damage or injury. We looked at the times and dates of all incidents, presence of a red cross or other medical emblem, types of attacks and weapons, numbers of dead and wounded, resulting damage to a hospital and where damage took place, and the amount of time it was closed or had limited services. For targeted attacks on personnel and patients, we analyzed the type of attack and resulting injuries, deaths, and disabilities. We also compared hospital location and size to the frequency of attacks and the resulting damage to the facility and related casualties. Pearson chi-squared analysis was conducted to identify risk differences between groups throughout the analysis. This test was chosen because of its relevance to unpaired categorical data. We also attempted to understand challenges in using the mobile systematic data collection tool and reasons that data collectors did not use the mechanism for all incidents. We conducted analysis of the data using Stata v 14.4 (http://www.stata.com).

### Comparing incidents

We compared attacks that SAMS collected with incidents confirmed by PHR in 2016 [26]. This analysis only included attacks on health facilities because PHR’s database does not include data on the other domains. SAMS and PHR use different methods to identify and verify attacks on health facilities. While SAMS uses its own data collectors, field personnel, and data managers, PHR uses open sources such as media reports and social media posts to identify incidents. There are differences in the geographical scope and the methods of collection in PHR’s and SAMS’ datasets. PHR collects data on health facilities across all governorates in Syria, whereas the SAMS data came from only four northern governorates that were at least partially controlled by opposition groups. Another difference is that SAMS data collectors focused on all four domains of attack, including targeted attacks on individuals and healthcare personnel independent of an attack on a health facility or transport, whereas the PHR database does not include direct attacks on transports, personnel, or patients. While comparison of the databases has limits (highly destructive incidents or those on larger facilities may have a higher probability of capture than less-damaging incidents), we hoped to put the SAMS data in the context of the current publicly available data on attacks on health.

PHR identifies and confirms incidents of attacks on health facilities using information from open sources, including, “United Nations, governmental, news agency, and non-governmental organization reports; journal articles; dissertations; social media and video sites; blogs; TV news footage; and reports produced inside Syria from the government or non-state armed groups,” as well as using information from field sources in Syria and the region [36]. PHR also employs a verification system that requires at least three independent sources to report on an incident in detail in order to corroborate each incident. PHR notes that because of their process, the number of incidents they report on is likely an underestimation of the total attacks on health facilities; in 2016, PHR only corroborated one-third of the reports of attacks on medical facilities they received and investigated (personal communication, PHR Research Staff Marianne Mollman, 2017). Only PHR’s confirmed reports were compared to attacks on facilities in the SAMS database and matched based on the following elements: (1) date, (2) location, and (3) name of healthcare facility. Investigators also scrutinized the type of attack and narrative details when this information was unclear or there were discrepancies between reports from PHR and data collected by SAMS. All documented incidents were categorized as follows:

- Definite match: If the date of attack, location of attack, and name of facility match. We allowed for 24-hour variation in date as might be attributed to a reporting delay.
- Probable match: If two out of the three (date, location, and name) matched. If the date was not an exact match, it needed to be within one week of the other.
- Possible match: If location or name of the hospital matched and the date was within one week of each other.
- Unique incident: Any incidents that do not match into any of the above category.

We considered utilizing the capture–recapture analysis to calculate an estimate of the total number of incidents [39,40]. However, given the practical challenges of ensuring that both databases are completely independent and identifying all possible colinearity among the databases, capture–recapture calculation of the total number of estimated incidents based on these databases has significant limitations and is not presented.

### Human subjects review

SAMS collected data as part of its ongoing work to provide health services to people in Syria. The Johns Hopkins Bloomberg School of Public Health Institutional Review Board approved analysis of secondary data. To guarantee the confidentiality of data collectors and protect the identity of the facilities, no individual identifiers, names, or specific locations are used in this analysis.

## Results

SAMS recorded 200 incidents of attacks on healthcare in 2016. Of those, 102 (51.0%) had completed reports on the Magpi application that allowed for more in-depth analysis. The attacks took place in the four northern governorates of Syria: Aleppo (*n* = 122), Idleb (*n* = 49), Homs (*n* = 17), and Hama (*n* = 12) from January 1, 2016, to December 31, 2016 (Table 1). Of these, we note that the majority involved aerial bombing of hospitals (91.5%; *n* = 183, including chemical bombs [*n* = 7]). Sixty-one percent (*n* = 122) of all attacks took place in Aleppo Governorate, of which 66.4%(*n* = 81) took place in Aleppo City.

**Table 1:** Geographical distribution (%) of incidents by governorate.

Governorate	Incidents	Casualties (deaths + injuries)
**Hama**	12 (6%)	12 (1.8%)
**Homs**	17 (8.5%)	45 (6.7%)
**Aleppo**	122(61%)	428 (63.5%)
**Idleb**	49(24.5%)	189 (28.0%)

### All attacks on health

**Fig 1:**
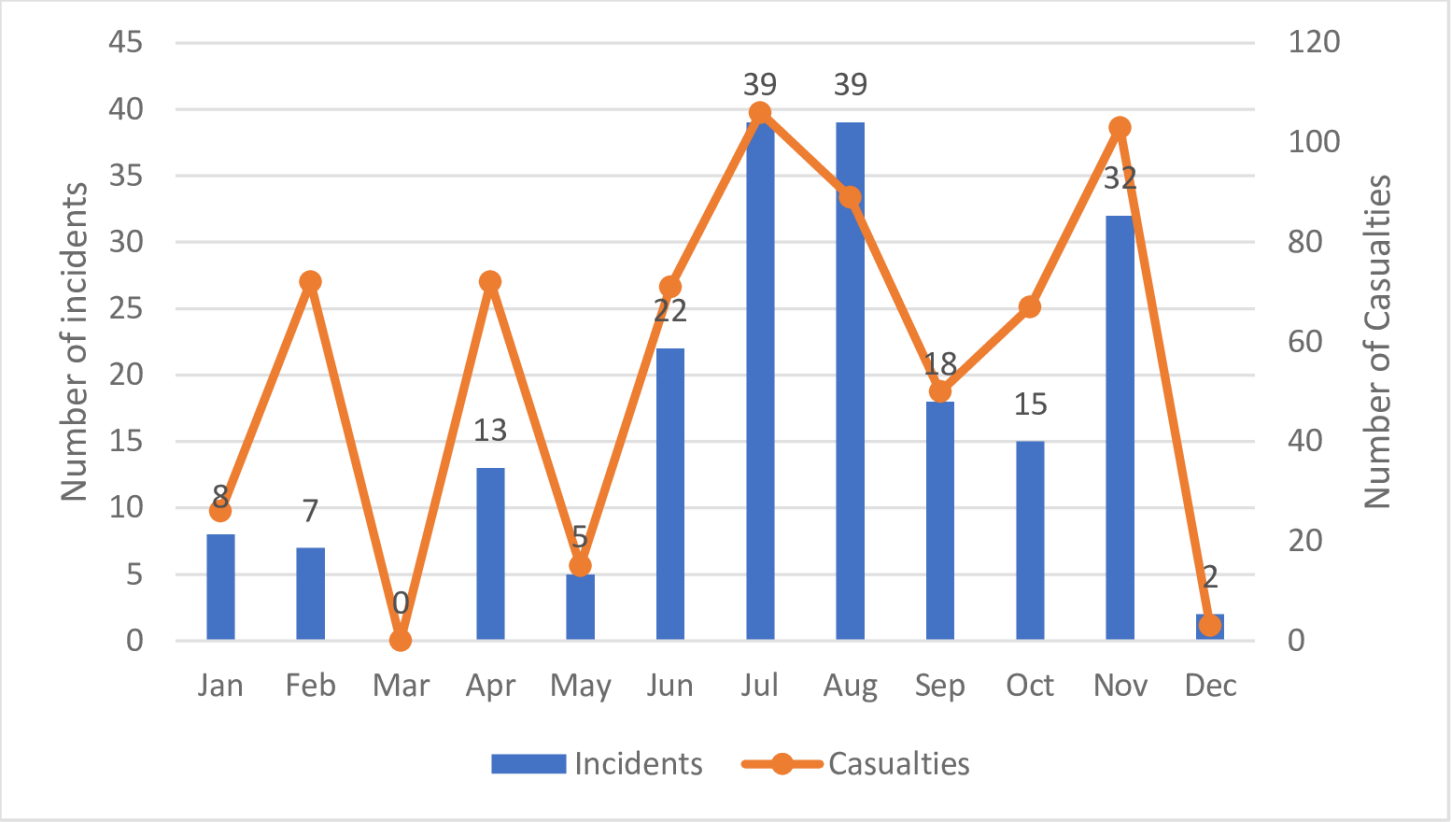
Number of incidents and casualties documented by SAMS (*N* = 200) by month (2016).

We found attacks on all four domains within the SAMS database: facilities, transports, personnel, and patients. There were 176 incidents that primarily involved healthcare facilities, 16 that primarily involved transports and eight that primarily involved personnel. At least one attack was documented in every month except March 2016 (Fig 1). We note that there was significant overlap among the domains: Within the 176 attacks on facilities, there were 69 that involved patients, 56 that involved personnel, and 44 that involved transports. There were no reports of direct targeting of patients in the data, though patients were injured and wounded in attacks on facilities and transports. Although we note that there were significant civilian casualties reported with nearly all of these attacks, our focus was limited to attacks on health systems, so we did not collect data on civilian injuries and deaths outside of healthcare personnel and patients present at the time of the attack. Overall, 297 deaths of healthcare professionals (37.7%; *n* = 112) and patients (62.3%; *n* = 185) were documented. An additional 433 injuries were documented among healthcare personnel (21.7%; *n* = 94) and patients (78.3%; *n* = 339). Overall, 30.0% (*n* = 60) of the incidents resulted in at least one injury and 34.5% (*n* = 69) in at least one death. Of the 90 incidents that involved at least one casualty (death or injury), the average number of deaths was 3.3 per incident, and 4.8 persons were injured per incident (including both healthcare personnel and patients).

#### Attacks on personnel

There were eight incidents in which healthcare personnel were attacked independent of strikes on facilities or transports. Among the personnel targeted, five incidents involved aerial bombs that hit physicians or other staff outside a hospital and one incident involved a physician who was shot by snipers and another killed by a landmine explosion. One incident involved the targeting of staff with chlorine gas.

#### Attacks on facilities and transports

Of the 176 incidents involving healthcare facilities, the majority (*n* = 120, 68.2%) involved hospitals. Outpatient clinics (*n* = 25), medical points (*n* = 4), and medical education facilities (nursing or medical schools, *n* = 3) were also attacked. Ancillary facilities (*n* = 24) included forensic centers, blood banks, specialty centers for physical therapy or preventative care, and medical administration (Table 2).

**Table 2:** Type of medical domain attacked (*n* = 200).

Domain	*Sub-Domain*	Attacks (percentage)
**Health Facilities**	*Hospital*	120 (60)
	*Clinic*	25 (12.5)
	*Medical Point*	4 (2)
	*Ancillary Services*	24 (12)
**Health Transports**		16 (8)
**Health Personnel**		8 (4)

Ninety-four distinct facilities were attacked, of which 33 (35.0%) were attacked more than once in 2016 alone, accounting for 115 of the 176 attacks on facilities. Hospitals attacked more than once averaged 3.5 attacks in 2016. Two health facilities were attacked more than 10 times; both were large hospitals in Aleppo City. Hospitals were significantly more likely to be attacked more than once than other types of facilities (*p* < 0.01).

Sixteen of the recorded incidents targeted health transports. Fourteen of these targeted ambulances, and two were attacks on convoys. Most of the attacks on transports (87.5%; *n* = 14) were the result of aerial bombs. One ambulance was shelled by ground artillery, and another was physically attacked and looted.

### Systematic survey analysis

We conducted an in-depth analysis of additional incident information gathered using the 102 incidents collected on the mobile systematic data collection tool. While the reasons for not utilizing the standardized tool for all incidents were not quantitatively addressed as part of this research, qualitative interviews with the field data collectors and data managers suggested a few challenges. They noted that the questionnaire on the mobile phone application took more time (approximately 15 minutes) than recording basic information, and the platform itself proved more challenging to use than we anticipated. They also noted that the use of a phone to document data appeared unfamiliar and disconcerting to some of the interviewees, who preferred a conversation to answering standardized questions.

Based on Pearson chi-squared testing, there were no notable differences in the domain (*p* = 0.995), location (*p* = 0.075), type of attack (*p* = 0.995), and total casualties (*p* = 0.08) between those attacks that were documented using the tool and those that were reported using the text messaging system. Of the 102 attacks recorded using the data collection tool, 90 were primarily attacks on facilities, eight on transports, and four on personnel. We found that data collectors spoke directly with healthcare personnel in 16 incidents, as well as other victims and/or witnesses who were not clinicians (usually hospital administrators) in 86 of the incidents.

We ascertained the level of damage resulting from the attacks as well as the ability of facilities and transports to function afterwards. Eighty-nine percent (*n* = 80) of the 90 health facilities were damaged (major damage: 37.5%; moderate damage: 27.5%; minimal damage: 35.0%) as a result of the attack. Forty-nine facilities (54.4%) were forced to close for various lengths of time (11 were forced to close for two weeks or more and eight were closed permanently). Attacks that caused major damage did not have significantly more casualties (*p* < 0.349) compared to those with moderate and minimum damage. However, facilities with major damage were significantly more at risk of being closed (*p* < 0.001). Fourteen percent (*n* = 13) of facilities had a red cross, red crescent, or other medical emblem; there was no significant difference in repeated attacks (*p* = 0.208) or probability of closure (*p* = 0.558) between hospitals with and without emblems.

The mobile data-collecting platform also allowed for more nuanced understanding of overlapping domains of attack. Although there were 16 documented attacks primarily on transports in the full dataset and eight attacks primarily on transports in the systematic data collection subset, there were a total of 35 incidents involving attacks on transports within the 102 incidents in the subset analysis. Thirty-four of 35 of these involved aerial attacks on the transports (the remaining was secondary to artillery). Ten of these attacks (28.6%) occurred while the ambulance was in transit. Eighty-six percent (*n* = 30) of the ambulances were taken out of service as a result of the attack, including 37.1% (*n* = 13) that were permanently destroyed. Eleven (31.4%) of the 35 ambulance systems displayed emblems.

As part of the analysis, we assessed various risk factors for increased frequency of attack, increased damage, injury, or death. We found that 27.0% of the documented attacks on facilities were on large tertiary care centers. These major hospitals were significantly more likely to be attacked more than once, as compared to other hospitals and clinics (average of 3.3 attacks versus 1.5 attacks, *p* = 0.026 utilizing the unpaired *t* test statistic). Although the incidents recorded occurred at all times of the day, a significantly higher proportion (37·0%; *p* < 0.01) occurred in the early morning hours, specifically between the hours of 05:00 and 08:00. We investigated differences in deaths (*p* = 0.149), presence of damage(*p* = .589), extent of damage (*p* = .363), and resulting closure between type of facility (*p* = .246). The only significant difference was among number of injuries (*p* < 0.002), but overall, there was no significant difference between facility types in these outcomes (Table 3).

**Table 3:** Comparisons of consequences between types of facility.

		Type of Facility				
		Field Hospital number (percentage)	Primary Health Center number (percentage)	Other number (percentage)	Total number (percentage)	*P* value (chi-squared)
**Incidents with Injuries**	*Yes*	16 (45.7)	37 (80.4)	12 (80)	65 (67.7)	0.002
	*No*	19 (54.3)	9 (19.6)	3 (20)	31 (32.3)	
**Incidents with Deaths**	*Yes*	19 (54.3)	34 (73.9)	11 (73.3)	64 (66.7)	0.149
	*No*	16 (45.7)	12 (26.1)	4 (26.7)	32 (33.3)	
**Incidents Damaging Facilities**	*Yes*	2 (5.7)	5 (10.9)	2 (14.3)	9 (9.5)	0.589
	*No*	33 (94.3)	41 (89.1)	12 (85.7)	86 (90.5)	
**Extent of Damage**	*Minimum*	12 (36.4)	19 (46.3)	2 (16.7)	33 (38.5)	0.363
	*Moderate*	11 (33.3)	14 (34.2)	5 (41.7)	30 (34.9)	
	*Major*	10 (30.3)	8 (19.5)	5 (41.7)	23 (26.7)	
**Forced To Close**	*Yes*	13 (37.1)	24 (55.8)	6 (42.9)	43 (46.7)	0.246
	*No*	22 (62.9)	19 (44.2)	8 (57.1)	49 (53.3)	

### Incident comparison

We compared incidents at facilities to a database of confirmed attacks from PHR. We only included verified incidents from the PHR database to ensure that we presented analysis using the highest quality of data. For our comparative analysis, we only used incidents that met the following criteria: (1) in the four northern governorates, (2) involving attacks on facilities, and (3) confirmed PHR reports. Excluding incidents that did not fit this criteria, we identified 89 attacks confirmed by PHR and 177 reported by SAMS data collectors in 2016 (one incident that was documented primarily as a transport attack in the SAMS database was reported as a confirmed attack on a facility and a transport system in the PHR database). In one case, an incident in the PHR database was documented as two separate attacks (on a hospital and on an affiliated administrative complex with health workers). We therefore adjusted the PHR incident number to 90 be conservative (Table 4). We were able to identify 52 definite matches and 8 probable matches among the databases. There were 30 unique incidents in the PHR dataset that were not available in the SAMS dataset and 117 in the SAMS dataset that were not found in the PHR dataset. Because of the limitations in avoiding colinearity among the databases, we did not calculate an estimated total number of possible incidents.

**Table 4:** Incident comparison between PHR and SAMS, 2016.

Organization	Documented Incidents (2016)	Noncomparable incidents	Comparable Incidents (on facilities), by Classification			
			*Relevant Incidents*	*Definite match*	*Probable Match*	*Unique Incident*
**PHR**	108	19	90*	52*	8	30
**SAMS**	200	23	177**			117

* One incident was counted as a single incident by PHR and as two separate incidents by SAMS

** One incident was counted as a transport attack by SAMS but reported as a facility attack by PHR

**Abbreviations:** PHR, Physicians for Human Rights; SAMS, Syrian American Medical Society.

## Discussion

This study confirms the large number of attacks on health, particularly on health facilities, consistent with both PHR’s and WHO/Health Cluster findings for the period covered. The 200 attacks SAMS found in a region with fewer than 200 hospitals is a tragic testament to the long-term destruction of the health system in Syria. Using a prospective surveillance methodology, we found that most of the incidents impacted multiple domains; killing or injuring patients and personnel as well as destroying transport capacities while targeting a hospital was frequent. We also found that the personnel and patient casualties consistently rose and fell with the frequency of hospital attacks over time. During the period covered, hospitals in the city of Aleppo suffered a significant portion of the attacks. Repeat attacks on hospitals were frequent and highlight the vulnerability of these facilities, especially larger centers, to bombardment, possibly because they are easily visualized and identified. Many other types of facilities, such as medical schools and blood banks, were also attacked or destroyed. While we identified primarily infrastructure attacks, many staff and patients were also killed or injured. On average, more than three people were killed in each incident involving at least one casualty.

These findings suggest that the number and severity of attacks on healthcare infrastructure and personnel in Syria are the most extreme example of a disturbing worldwide phenomenon. In 2016 alone, health facilities and health workers were attacked or subject to obstruction or interference in at least 23 countries in conflict [1]. Attacks on healthcare globally have not been systematically tracked, so comparisons between Syria and other areas of conflict cannot be made with certainty. However, regardless of methodology used, reports of attacks in dozens of countries over the past two decades reveal no other place in the world where so many hundreds of hospitals have been attacked as has occurred in Syria [1,7]. Sound documentation of attacks on and obstruction and interference with healthcare is essential to understand the nature of the violence; causes for health worker migration from conflict zones; the short-, intermediate-, and long-term consequences of such attacks on the health system; the potential means of preventing such attacks; and to build political will to stop the attacks and bring perpetrators to justice [2].

### Systematic data collection

There is wide agreement that sound methods to track attacks on and interference with healthcare facilities and personnel are essential. In collaboration with SAMS, we developed a standardized questionnaire for reporting attacks on healthcare in Syria that complements other methods for documenting attacks developed by PHR and by the WHO health cluster for northern Syria. Using the questionnaire, SAMS reported 102 attacks in the northern governorates of Syria, but our analysis of all of SAMS’ incident data revealed that the tool was not used in nearly half of the reported incidents. This experience suggests that it is challenging to utilize a systematic, field-based data collection mechanism consistently during times of intense conflict but that, when it is used, it provides valuable, detailed information that can inform advocacy and policy. We highlight that use of the data collection tool permitted comparisons in the attacks based on size and type of health facility, presence of an emblem, operational capacity after the attack, and source of information. It allowed for more nuanced information on attacks that involved multiple domains of health services. However, use of the tool was considered lengthy and time consuming by some of the field data collectors. Use of this tool was feasible in this context, but to be used for each incident, we believe it requires a relatively high level of administrative, technological, and epidemiological support. We also believe that simplification of the questionnaire would be helpful, even at the cost of the absence of data collection on certain topics. The research team has reviewed these concerns and is prepared to modify and revise the tool to achieve the right balance between comprehensiveness and ease of use.

Using systematic data collection, we found that there is a significantly increased risk of multiple attacks on larger tertiary care hospitals. A more nuanced understanding of the damage and destruction caused by these attacks is important in order to better gauge the health services that these communities might require going forward. We note that only a small percentage of facilities and transports had a medical emblem, suggesting in a context where emblems historically have protected from attack that they may be perceived to represent a vulnerability in Syria. Similarly, the massive scale of attacks on hospitals in particular may represent the ease of distinguishing these structures from the air and highlight the paradoxical humanitarian framework at play in Syria, where signs of humanitarian operations may actually be a risk for attack.

### Incident comparison

In collaboration with PHR, our findings also suggest that no one methodology currently used to document attacks in Syria is sufficient to identify the full scope of the assaults. SAMS documented 177 attacks on health facilities, and PHR documented 90 confirmed incidents in the comparable geographical region and time frame, but PHR received reports of at least 234 relevant incidents in its aggregate database that includes both verified and unverified attacks. The reasons for the difference is likely a product of the limitations of the methods used. For on-the-ground reporting, such as that used by SAMS, the limitations are likely the inability to access all areas to take reports; for the primarily open-source reporting used by PHR, the limitations are likely the result of lack of media reporting of all incidents, as well as the high standard for corroboration required by PHR. By matching the incidents, we found that only 60 of the incidents were matched between the two datasets. Thirty of the PHR incidents and 117 of the SAMS incidents were not found in the other dataset. These figures highlight the difficulty in capturing all incidents against healthcare in a challenging humanitarian context and illustrate the attacks that are likely being omitted by any single data collection mechanism. Collaborations among partners can provide in-depth detail while also helping to establish the full breadth of the attacks. The UN Health Cluster for northern Syria, of which SAMS is a member, utilizes such a collective methodology and has reported 158 verified incidents between November 2015 and December 2016 [23].

A subsidiary finding is that the data collection program appears to have empowered local communities with whom data collectors interacted to contribute to the documentation of incidents. This type of on-the-ground system can potentially help energize communities, present useful, real-time data, and contribute to advocacy and diplomatic channels in the context of some quantitative data. Our colleagues at SAMS have used this data to inform their advocacy and programming efforts.

### Limitations

The study has some important limitations. We utilized a previously validated survey tool based in international humanitarian law to develop a standardized approach to documenting attacks on health globally. However, all conflicts are unique, and further work may be needed to adapt the tool to capture attacks in other settings.

Documentation in this challenging, complex, and violent context of civil conflict is limited by the accessibility of the hospitals and the willingness of staff, victims, and witnesses to discuss their experiences, leading to under- or over-reporting. SAMS utilized data collectors who live and work among the communities where they documented attacks. While this familiarity with the culture and community may have mitigated any hesitancy to discuss violence, witnesses may still have been reluctant to share information due to personal and/or security risks. Additionally, the data collectors were limited in their ability to document attacks during times of high risk to their own safety or when excessive attacks in a short time frame would limit their ability to visit various incident sites. We note that while reports from major incidents or attacks on larger facilities may have been more rapidly transmitted than those on smaller facilities, data collectors and SAMS staff made great efforts to ensure that attacks on smaller facilities and individuals were regularly surveilled.

The data on the overall number of attacks as reported by SAMS come from two different collection methods: the simple reporting of key details about an event by text messaging and more in-depth information about incidents available through the systematic data collection tool. It is possible that there were qualitative differences between the attacks reported via the systematic data collection tool and those reported elsewhere. In our review of the underlying incidents, we did not identify any relevant patterns in how a particular type of event was reported; however, it is possible that they exist. The use of mobile technology, though it rendered more details about specific incidents, was not used in about half of the incidents, suggesting that the program will require further revisions and training to ensure that it is used more consistently. Sporadic interruptions of cellular reception and internet connectivity were also reported to limit use of mobile data collection and transmission.

We note that this program did not include a formal system for secondary or tertiary verification of incidents. However, SAMS data managers utilized a variety of methods to ensure data quality, including checking all incidents among the health cluster network, corroborating with other field staff and health personnel, communicating back and forth with data collectors, and gathering photographic and video evidence to ensure data quality. While there may be a role for the larger health cluster to use formal mechanisms and external reports to verify each incident, SAMS’ role was to create a reliable and accurate system of original reporting. Nevertheless, the findings of this study are based on reports that have been extensively checked and authenticated. We did not discern any differences in verification between Magpi-reported incidents and others.

Because of the frequency of bombing and shelling of health facilities, the majority of our data involve such events. While we documented some incidents of attacks on personnel independent of facility attacks, our data likely understate these assaults, as well as obstructions of access to care, since they may garner limited attention in comparison to incendiary strikes. The survey tool was developed to document attacks across all domains, which may be more frequently utilized in other violent conflicts. There is also a risk of reporting bias. Although data collectors documented attacks within hours or, at most, a few days of an attack, there may be recall bias or other reporting bias in the surveys. Attacks on larger health facilities may be more likely to be reported, while attacks on smaller facilities may be underreported, potentially because there is less impact or fewer staff or witnesses to report the incident. Another source of possible bias is in the political views of data collectors and witnesses. In almost all cases, photographic evidence and multiple victim corroborations have assisted in mitigating the likelihood of bias.

We evaluated the SAMS and PHR data for independence in their methodologies. SAMS staff does not coordinate with PHR for its data collection or vice versa. PHR’s data was retrospectively reviewed, as they do not have any staff on the ground in Syria and utilize social and news media accounts of attacks. SAMS data collectors arrive at the scene of the attack and use nonmedia means of obtaining information on attacks on health, including eyewitness accounts, networking with administrators and health staff, and private SAMS messaging groups. Ideally, our comparative analysis would have utilized more than one partner, but given the constraints of setting, limited collection of this type of data, and the challenges in collecting detailed information on attacks, we believe our approach offers the most comprehensive understanding of attacks on healthcare in Syria to date.

### Conclusion

This study shows the potential contribution of employing field data collectors in documenting attacks on healthcare. Along with reporting by health cluster organizations (as WHO has shown) and analysis of photographic, video, media, social media, and other materials (as PHR has done), dedicated field data collectors can collect direct and powerful evidence of attacks. Standardized questions can yield detailed, consistent information, and the use of a mobile application can allow secure and quick transmission of data.

Tracking attacks on healthcare is vitally important for three purposes. First, sound data about attacks is essential to help the global community know exactly what attacks are taking place, where they are taking place, what their impacts are, and, in some cases, who the perpetrator is. Second, it is essential to stimulate action at the international level that can prevent future attacks, promote investigations, and ultimately hold perpetrators accountable. Third, it can assist organizations in designing harm mitigation strategies, as SAMS has previously attempted [41]. The ongoing attacks on healthcare in Syria illustrate how critical it is to use every mechanism possible to develop political will to stop the attacks, conduct investigations, and prosecute those who commit these abuses and violate international law.

The analysis also suggests a far higher level of attacks on healthcare in Syria than previously reported. We highlight that, beyond the violent injuries that are documented in this manuscript, there must be an informed discussion of the consequence for mortality and morbidity that results from the destruction of the healthcare infrastructure, including from communicable diseases, lack of preventative care, exacerbations of chronic conditions, limited-capacity prevention programs, and the fear of seeking out healthcare services when they are seen as a target for attack.

This study also demonstrates the capacity of locally based organizations and data collectors, in collaboration with physicians and other health staff, to contribute to documentation of attacks on health using a mobile systematic data collection application and act as potent agents to report and chronicle this violence.

The UN Security Council, as part of Resolution 2286 passed on May 3, 2016, “condemns acts of violence, attacks and threats against the wounded and sick, medical personnel and humanitarian personnel … as well as hospitals and other facilities and demands that all parties of conflicts comply with their obligations under international law” [13]. WHO is mandated to “provide leadership at the global level in developing methods for systematic collection and dissemination of data on attacks” [15]. WHO is beginning implementation of its global system, and as its methodology develops, it can consider the uses of field data collectors as part of meeting the enormous challenges in standardizing data collection methods for attacks on healthcare. Additionally, NGOs and other entities in conflicts where healthcare is under attack can use the questionnaire and mobile application and field data collectors to gain data about the attacks. We hope that the development of this tool and the reporting that results will assist in setting a course to secure compliance with international law and end impunity.

## Supporting information

S1 Fig Example of Magpi data entry platform on mobile phone. (TIF)

S1 Data Deidentified database of analyzed events. (XLSX)

## Abbreviations

NGO: nongovernmental organization
PHR: Physicians for Human Rights
SAMS: Syrian American Medical Society
WHO: World Health Organization
