# Supplementary figures and images for: Determining the scope of attacks on health in four governorates of Syria in 2016: Results of a field surveillance program
Source: PLoS Med. 2018 Apr 24;15(4):e1002559. doi: 10.1371/journal.pmed.1002559 (PMC5915680; doi:10.1371/journal.pmed.1002559)

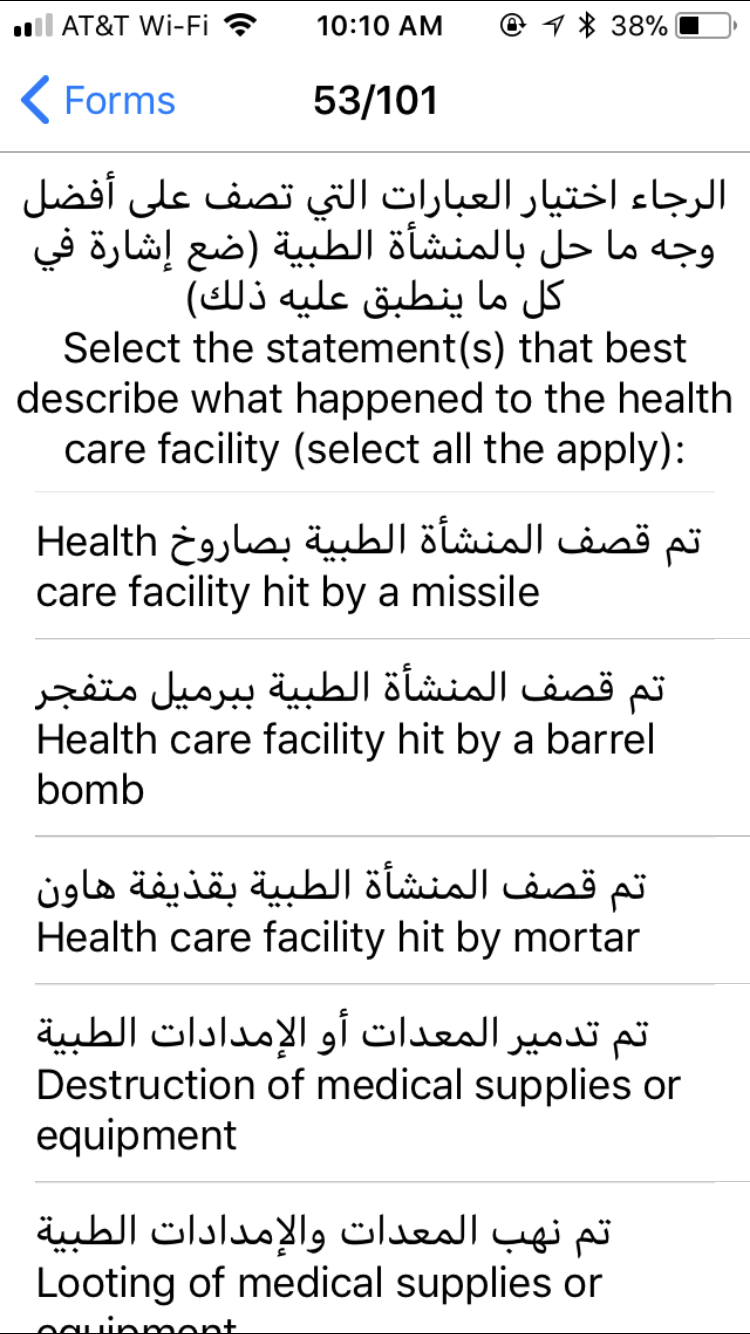

Supplement: S1 Fig — (TIF) [file pmed.1002559.s001.tif]
